# TNFα stimulates osteoclastogenesis and expression of CX3CL1 in non-adherent bone marrow cells

**DOI:** 10.1016/j.bbrep.2025.102155

**Published:** 2025-07-11

**Authors:** Yuto Otsuka, Narumi Hattori, Hiromasa Aoki, Kohki Toriuchi, Yasumichi Inoue, Hidetoshi Hayashi, Gen Kuroyanagi, Yohei Kawaguchi, Yuko Waguri-Nagaya, Mineyoshi Aoyama

**Affiliations:** a Department of Pathobiology, Nagoya City University Graduate School of Pharmaceutical Sciences, 3-1 Tanabe-dori, Mizuho-ku, Nagoya, Aichi 467-8603, Japan; b Department of Cell Signaling, Nagoya City University Graduate School of Pharmaceutical Sciences, 3-1 Tanabe-dori, Mizuho-ku, Nagoya, Aichi 467-8603, Japan; c Department of Innovative Therapeutic Sciences, Cooperative Major in Nanopharmaceutical Sciences, Nagoya City University Graduate School of Pharmaceutical Sciences, 3-1 Tanabe-dori, Mizuho-ku, Nagoya, Aichi, 467-8603, Japan; d Department of Orthopaedic Surgery, Nagoya City University Graduate School of Medical Sciences, 1 Kawasumi, Mizuho-cho, Mizuho-ku, Nagoya, Aichi 467-8601, Japan

**Keywords:** Osteoclast, TNFα, Chemokine, Microenvironment, Rheumatoid arthritis

## Abstract

The balance between bone formation by osteoblasts and bone resorption by osteoclasts is a critical step in maintaining bone homeostasis. Excessive activation of osteoclasts is important in the bone destruction seen in diseases such as osteoporosis and rheumatoid arthritis. The microenvironment around bone marrow cells regulates osteoclastogenesis through cytokine expression. Tumor necrosis factor–α (TNFα) is a proinflammatory cytokine that plays an important role in bone loss in rheumatoid arthritis. Therefore, this study investigated the effect of TNFα on osteoclastogenesis via chemokines produced by microenvironmental cells. In *in vitro* culture of mouse bone marrow cells, TNFα added simultaneously with receptor activator of nuclear factor kappa-B ligand (RANKL) and macrophage colony-stimulating factor (M-CSF) from the initiation of culture significantly inhibited osteoclast formation. By contrast, TNFα stimulation from 7 days after prior stimulation with RANKL and M-CSF significantly promoted osteoclastogenesis. This late-stage promotional effect was associated with the upregulation in non-adherent bone marrow cells of C-X3-C motif chemokine ligand 1 (CX3CL1) and C-X-C motif ligand 7 (CXCL7), which are potent chemoattractant and adhesion molecules. Neutralizing antibodies against TNF receptor 1 (TNFR1) and TNFR2 markedly suppressed osteoclastogenesis in the presence of TNFα. Additionally, these antibodies significantly reduced CX3CL1—but not CXCL7—mRNA expression levels in non-adherent bone marrow cells. In conclusion, our results suggest that TNFα treatment in the late stage promotes osteoclast formation and increases the expression of CX3CL1 in non-adherent bone marrow cells. These findings highlight the time-dependent role of TNFα in osteoclastogenesis relative to non-adherent bone marrow cells.

## Highlights

• TNFα exerts a time-dependent, biphasic effect on osteoclastogenesis.
• Non-adherent bone marrow cells regulate late-stage osteoclastogenesis via TNFα.
• CX3CL1 expression by non-adherent bone marrow cells promotes osteoclastogenesis.
• Non-adherent bone marrow cells are a therapeutic target in inflammatory bone loss.

## Abbreviations:

αMEM: alpha minimum essential medium
CX3CL1: C-X3-C motif chemokine ligand 1
CX3CR1: chemokine (C-X3-C motif) receptor 1
CXCL7: C-X-C motif ligand 7
CXCR2: chemokine (C-X-C motif) receptor 2
DC-STAMP: dendritic cell–specific transmembrane protein
EDTA: ethylenediaminetetraacetic acid
FBS: fetal bovine serum
IGF2: insulin-like growth factor 2
IL-1β: interleukin 1–beta
IL-6: interleukin 6
iNOS: inducible nitric oxide synthase
M-CSF: macrophage colony-stimulating factor
NFATc1: nuclear factor of activated T cells 1
OPG: osteoprotegerin
PBS: phosphate-buffered saline
qPCR: quantitative polymerase chain reaction
RANKL: receptor activator of nuclear factor kappa-B ligand
RT-PCR: reverse transcription–polymerase chain reaction
SDF1: stromal cell–derived factor 1
SEM: standard error of the mean
TNFα: tumor necrosis factor–alpha
TNFR1: tumor necrosis factor receptor 1
TNFR2: tumor necrosis factor receptor 2
TRAP: tartrate-resistant acid phosphatase

## 1 Introduction

To maintain bone homeostasis, an adequate balance between osteogenesis and osteoclastogenesis is critical [1,2]. Excessive osteoclast activation is associated with bone resorption in various metabolic and inflammatory bone diseases, including osteoporosis and bone erosion in rheumatoid arthritis [[3], [4], [5]]. Rheumatoid arthritis is an autoimmune disease characterized by the proliferation of synovial tissue, joint inflammation, and bone destruction. In the advanced stage of rheumatoid arthritis, both the articular cartilage and bone joints are damaged, leading to joint deformation and a decrease in the ability to engage in daily activities [[6], [7], [8]]. The expression of proinflammatory cytokines such as tumor necrosis factor–α (TNFα), interleukin-6 (IL-6), and IL-1β increases around inflamed joints, leading to osteoclast activation and joint destruction [[6], [7], [8]]. The significance of TNFα in bone loss is demonstrated by the efficacy of anti-TNFα therapies, which can substantially decrease inflammation and inhibit joint damage in rheumatoid arthritis treatment [9]. However, 20–40 % of patients with rheumatoid arthritis do not respond to TNFα inhibitors [9], necessitating more precise therapeutic strategies for TNF inhibition.

Osteoblasts differentiate from mesenchymal cells, whereas osteoclasts differentiate from bone marrow–localized cells such as macrophages and monocytes [10,11]. Osteoblastic cells strictly regulate the differentiation of osteoclasts through a variety of hormones and cytokines, including TNFα and IL-6 [12,13]. Osteoclast differentiation also requires receptor activator of nuclear factor-κB ligand (RANKL) secreted by osteoblasts in addition to macrophage colony-stimulating factor (M-CSF) [[12], [13], [14]]. Details regarding how inflammatory cytokines such as TNFα regulate osteoclastogenesis in rheumatoid arthritis have not yet been fully elucidated, however. It is particularly unclear how different types of cells form a microenvironment suitable for osteoclastogenesis via the TNFα signaling pathway [15]. Under physiological conditions, the balance between RANKL and its decoy receptor, osteoprotegerin (OPG), tightly regulates osteoclastogenesis to maintain bone integrity [16]. However, in rheumatoid arthritis, the inflammatory milieu shifts this balance by upregulating RANKL and downregulating OPG [17]. Activated synovial fibroblasts contribute to osteoclast formation by producing RANKL at joint erosion sites [18].

The microenvironmental niche formed by osteoblasts, synovial fibroblasts, T lymphocytes, and bone marrow stromal cells is important for osteoclastogenesis under several conditions, including hypoxia and inflammation [19,20]. In rheumatoid arthritis, this niche is profoundly altered by persistent inflammatory stimulation. The hypoxic and cytokine-rich environment within the synovium alters the phenotype of synovial fibroblasts, inducing them to secrete not only RANKL but also chemokines and various matrix-degrading enzymes that facilitate immune cell recruitment and osteoclast maturation [21]. Additionally, the structural remodeling of the synovial membrane disrupts normal stromal-immune interactions, further intensifying osteoclastogenic signaling [22]. Our previous studies highlighted the importance of this microenvironment, demonstrating that factors such as stromal cell–derived factor 1 (SDF1), C-X3-C motif chemokine ligand 1 (CX3CL1), and insulin-like growth factor 2 (IGF2), produced by non-osteoclastic supporting cells, promote osteoclastogenesis under conditions of hypoxia or IL-1β stimulation [[23], [24], [25], [26]]. Given that both hypoxia and IL-1β are prominent features of the rheumatoid synovial environment [27], these findings suggest a significant mechanistic link between inflammatory microenvironmental cues and pathological bone resorption.

TNFα is a proinflammatory cytokine produced by various types of cells, including macrophages, monocytes, endothelial cells, osteoblasts, and fibroblasts [19,28]. With regard to signaling, TNFα binds to two types of receptors, TNF receptor 1 (TNFR1) and TNFR2. TNFR1 is expressed across a wide variety of cell types, whereas TNFR2 is primarily expressed by specific cell populations, including bone marrow cells, regulatory T cells, and endothelial cells [28]. TNFα plays a pivotal role in the progression of rheumatoid arthritis [[6], [7], [8]], contributing to both synovial inflammation and joint destruction. Through TNFR1-mediated signaling, TNFα activates the NF-κB and mitogen-activated protein kinase pathways, resulting in the transcription of proinflammatory genes, RANKL expression, and survival signals in synovial and immune cells [[29], [30], [31]]. Although TNFR2 is less broadly expressed, it modulates immune cell activity and may play a role in tissue remodeling. This dual receptor system enables TNFα to exert both direct and indirect effects on bone resorption [32]. In non-adherent bone marrow cells, including osteoblasts and osteocytes, TNFα induces RANKL expression and regulates osteoclast-mediated bone resorption [33,34]. However, TNFα also reportedly acts directly on osteoclasts in the presence of RANKL to promote osteoclast formation [[35], [36], [37]]. Thus, TNFα facilitates osteoclastogenesis via multiple pathways. However, how TNFα regulates osteoclastogenesis in environments including non-adherent bone marrow cells is not completely understood.

In this study, we attempted to elucidate the precise role of non-adherent bone marrow cells in osteoclast formation in the presence of TNFα and evaluate the function of non-adherent bone marrow cells under conditions of TNFα exposure. The results of this investigation are expected to shed light on how bone marrow–resident supporting cells contribute to osteoclastogenesis in autoimmune diseases such as rheumatoid arthritis, in which TNFα plays a central role in pathological bone destruction.

## 2 Materials and methods

### 2.1 Mice and materials

This study used 6- to 8-week-old male C57BL/6 N mice (provided by Shizuoka Experimental Animal Center, Hamamatsu, Japan). The animal protocols were approved by the Institutional Animal Care and Use Committee (approval no.: H29–P-01). Recombinant human M-CSF was purchased from Kyowa Hakko Kogyo Co., Ltd. (Tokyo, Japan). Recombinant soluble human RANKL was prepared by Oriental Yeast Co., Ltd. (Tokyo, Japan). Recombinant mouse TNFα was purchased from Biolegend, Inc. (San Diego, CA, USA). Mouse anti-TNFR1 and anti-TNFR2 antibodies were purchased from Thermo Fisher Scientific, Inc. (Waltham, MA, USA).

### 2.2 Osteoclast formation

Bone marrow cells were prepared from the tibias and femurs of C57BL/6 N mice as previously described [26]. The cells were harvested in alpha minimum essential medium (αMEM) (FUJIFILM Wako Pure Chemical Corp., Osaka, Japan) containing 10 % fetal bovine serum (FBS) and penicillin/streptomycin and maintained in a humidified atmosphere with 5 % CO_2_. Cultured cells were seeded at 2.5 × 10^6^ cells/well in a 48-well plate in αMEM containing 10 % FBS. After 24 h, half of the medium was replaced and supplemented with 20 ng/mL M-CSF and 100 ng/mL RANKL for osteoclast culture. The medium was changed every 3 days, with replacement of half of the old medium with an equal volume of fresh medium. The cells were cultured for a total of 9 days and used for experiments.

### 2.3 Tartrate-resistant acid phosphatase (TRAP) staining

After stimulation of osteoclast differentiation using RANKL and M-CSF, the cells were rinsed once with phosphate-buffered saline (PBS) and fixed by incubation in 10 % formaldehyde for 10 min. Subsequently, the cells were washed twice with PBS and stained with TRAP solution containing 0.05 M acetate buffer, 0.03 M sodium tartrate, 0.1 mg/mL naphthol AS MX phosphate disodium salt, and 0.3 mg/mL Fast Red for 30 min at 37 °C. The number of multinucleated TRAP-positive cells was then determined using a microscope at 40 × to 100 × magnification (Olympus Co., Tokyo, Japan), and the cells were traced using the GNU Image Manipulation Program (GIMP; https://www.gimp.org; GIMP Development Team). The resulting images were quantified using ImageJ software (US National Institutes of Health, Bethesda, MD, USA).

### 2.4 Isolation of osteoclasts and non-adherent bone marrow cells by pronase treatment

After 9 days of culture with M-CSF and RANKL to induce osteoclast differentiation, two distinct cell populations were separated based on differential adherence to the culture dish following pronase treatment. The firmly adherent population was collected as the osteoclast-enriched fraction, whereas the floating or less adherent population was operationally defined in this study as non-adherent bone marrow cells. This separation method was adapted from protocols designed to purify osteoclasts by removing less adherent osteoblastic/stromal cells [23,38]. Briefly, after removing the culture supernatant, the adherent cell layer was washed once with PBS. Subsequently, the cells were treated with 1 mL of PBS containing 0.001 % pronase (Calbiochem, La Jolla, CA, USA) and 0.02 % ethylenediaminetetraacetic acid (EDTA) for 10 min at room temperature. This treatment selectively detaches less adherent cells. The supernatant containing detached cells was carefully collected as the microenvironment-forming cell fraction. The success of this separation step was confirmed visually under a phase-contrast microscope before collecting the fractions. The majority of smaller, mononuclear cells detached, but large multinuclear osteoclasts stayed firmly attached to the dish. The cells that remained firmly adherent to the culture dish after pronase-EDTA treatment were considered osteoclasts.

### 2.5 Reverse transcription–polymerase chain reaction (RT-PCR) analysis

Total RNA was extracted using RNAiso Plus (Takara Bio Inc., Otsu, Japan). Reverse transcription was performed from total RNA using a PrimeScript RT reagent kit (Perfect Real Time; Takara Bio Inc.). Real-time PCR was performed using GoTaq qPCR Master mix (Promega Co., Madison, WI, USA) and StepOnePlus (Applied Biosystems, Waltham, MA, USA). Amplification was performed by activation of GoTaq Hot Start Polymerase at 95 °C for 2 min, followed by amplification for 40 cycles at 95 °C for 15 s and 60 °C for 10 min. The relative quantification of target genes was normalized according to β-actin mRNA after confirming that cDNAs from different genes were amplified with the same efficiency. The primer sequences are presented in Table 1.

**Table 1:** Primer information for quantitative RT-PCR analysis.

Target gene	Forward primer (5′-3′)	Reverse primer (5′-3′)	GenBank accession no.
β-Actin	CTAAGGCCAACCGTGAAAAG	GTACGACCAGAGGCATACAG	NM_007393
RANKL (Tnfsf11)	GCAGCATCGCTCTGTTCCTGTA	CCTGCAGGAGTCAGGTAGTGTGTC	NM_011613
RANK (Tnfrsf11a)	TTCGTCCACAGACAAATGCAAAC	GCTGCAGACCACATCTGATTCC	NM_009399
IL-1β (Il1b)	TCCAGGATGAGGACATGAGCAC	GAACGTCACACACCAGCAGGTTA	NM_008361
IL-1RI (Il1r1)	GCTGACTTGAGGAGGCAGTTT	ATGAGCCCCAGTAGCACTTT	NM_008362
IL-1RII (Il1r2)	GATCCAGTCACAAGGGAGGA	CCAGGAGAACGTGGAAGAGA	NM_010555
SDF-1 (Cxcl12)	GCACTTTCACTCTCGGTCCA	GCGATGTGGCTCTCGAAGAA	NM_021704
CX3CL1	ACCTATGGCCCTGACATCATCAC	CTTGCCAGCCCTCAGAATCAC	NM_009142
CXCL7 (Ppbp)	GGAGTTCACTGTGCTGATGTGGA	CACAGATGAAGCAGCTGGTCAGTAA	NM_023785
CXCR4	GTTGCCATGGAACCGATCA	TGCCGACTATGCCAGTCAAGA	NM_009911
CX3CR1	GCAGTCTGTATGTTTGTGTCGAGGA	AGCATGGTGTTGCAATAACAAAGG	NM_009987
CXCR2	TCTGCTCACAAACAGCGTCGTA	GAGTGGCATGGGACAGCATC	NM_009909
IGF2	AGTTTGTCTGTTCGGACCGC	GGGGTATCTGGGGAAGTCGT	NM_010514
iNOS (Nos2)	ACCCTAAGAGTCACCAAAATGGC	TTGATCCTCACATACTGTGGACG	NM_010927
NFATc1	GGTAACTCTGTCTTTCTAACCTTAAGCTC	GTGATGACCCCAGCATGCACCAGTCACAG	NM_01679
DC-STAMP	TGTATCGGCTCATCTCCTCCAT	GACTCCTTGGGTTCCTTGCTT	NM_029422
OPG (Tnfrsf11b)	GGGACCAAAGTGAATGCCGA	CTGCTCTGTGGTGAGGTTCG	NM_008764.4

### 2.6 Statistical analysis

All statistical analyses were performed using EZR (Saitama Medical Center, Jichi Medical University, Saitama, Japan), a graphical user interface for R (The R Foundation for Statistical Computing, Vienna, Austria) [39]. One-way analysis of variance was used to compare differences in continuous data between three or more groups, followed by Tukey's post hoc test. Comparisons between two groups were assessed using Student's *t*-tests. Data are expressed as the mean ± SEM. A *p*-value of <0.05 was considered statistically significant.

## 3 Results

### 3.1 TNFα induced osteoclast formation at the late culture time point in the presence of RANKL and M-CSF

**Fig. 1:**
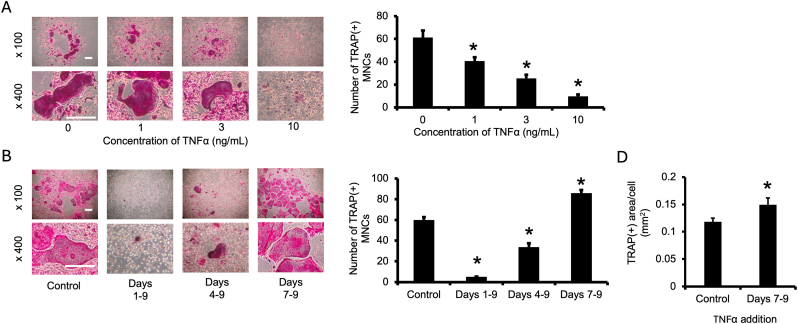
**TNFα induced osteoclast formation at the late culture time point in the presence of RANKL and M-CSF.** (A) Cultured bone marrow cells were treated with 100 ng/mL RANKL, 20 ng/mL M-CSF, and indicated doses of TNFα (1, 3, or 10 ng/mL) for 10 days. (B) Cultured bone marrow cells were treated with 100 ng/mL RANKL and 20 ng/mL M-CSF for 10 days. TNFα (10 ng/mL) was also added in the early (days 1–4), middle (days 4–9), and late (days 7–9) stages of cell culture. Cells were assessed by TRAP staining, and TRAP-positive multinuclear cells containing more than three nuclei (TRAP[+] MNCs) were counted. n = 8 per group, ∗*p* < 0.05 compared with the control. Images are shown at low ( × 100) and high ( × 400) magnification to visualize the overall cell density and detailed morphology of osteoclasts, respectively. Scale bars = 0.25 mm.

In this study, we first treated mouse bone marrow cells with TNFα in the presence of RANKL and M-CSF to examine the effect of TNFα on osteoclast formation. When cells were stimulated with RANKL and M-CSF in conjunction with TNFα from the start of the experiment, osteoclast formation was significantly reduced in a TNFα concentration–dependent manner (Fig. 1A). We next performed experiments in which TNFα was added in the early (days 1–4), middle (days 4–9), and late (days 7–9) stages of cell culture. Interestingly, the addition of TNFα beginning in the late stage of cell culture (days 7–9) strongly induced osteoclast formation (Fig. 1B). Furthermore, analysis of the average area of TRAP-positive multinucleated osteoclasts showed that the addition of TNFα in the late stage of cell culture also significantly increased the average area of TRAP-positive multinucleated osteoclasts (Fig. 1C).

### 3.2 TNFα mRNA expression level was higher in non-adherent bone marrow cells, whereas TNFR1 and TNFR2 mRNA expression levels were higher in osteoclasts

**Fig. 2:**
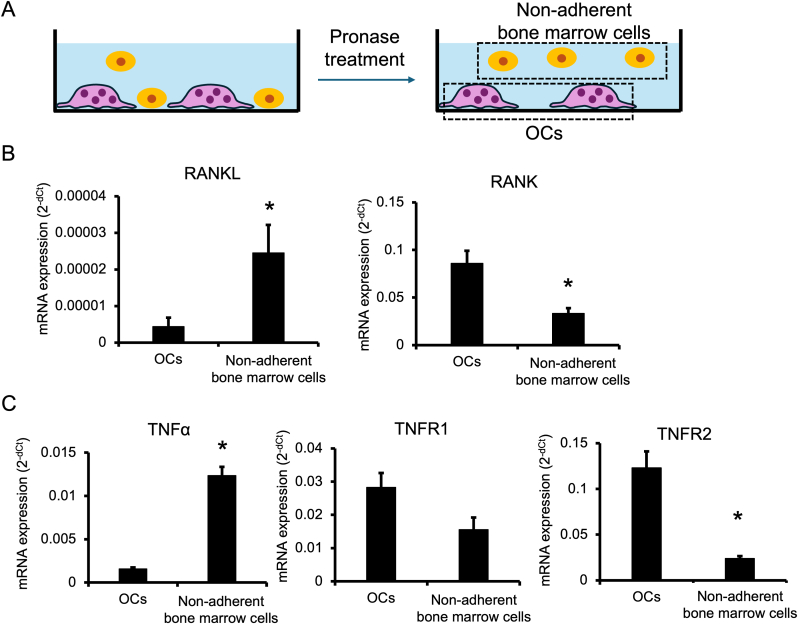
**TNFα mRNA expression level was higher in non-adherent bone marrow cells, whereas TNFR1 and TNFR2 mRNA expression levels were higher in osteoclasts.** Cultured bone marrow cells were treated with 100 ng/mL RANKL and 20 ng/mL M-CSF for 9 days. Cells were separated into osteoclasts and non-adherent bone marrow cells using the pronase procedure (A). RANKL and RANK (B), and TNFα, TNFR1, and TNFR2 (C) mRNA expression levels were quantified by quantitative RT-PCR. mRNA expression levels were normalized to that of β-actin mRNA. n = 3 per group, ∗*p* < 0.05 compared with osteoclasts.

We next performed quantitative RT-PCR analysis of mRNAs, including RANKL, RANK, TNFα, TNFR1, and TNFR2 from isolated osteoclasts and non-adherent bone marrow cells (Fig. 2A). The expression of RANKL mRNA was significantly higher in non-adherent bone marrow cells than in osteoclasts, whereas RANK mRNA expression was significantly higher in osteoclasts than in non-adherent bone marrow cells, indicating that osteoclasts and non-adherent bone marrow cells were well separated (Fig. 2B). Furthermore, the level of TNFα mRNA expression was higher in non-adherent bone marrow cells. TNFR1 and TNFR2 mRNA expression levels were higher in osteoclasts (Fig. 2C).

### 3.3 TNFα treatment in the later stage of culture increased CX3CL1 and CXCL7 mRNA expression levels in non-adherent bone marrow cells

**Fig. 3:**
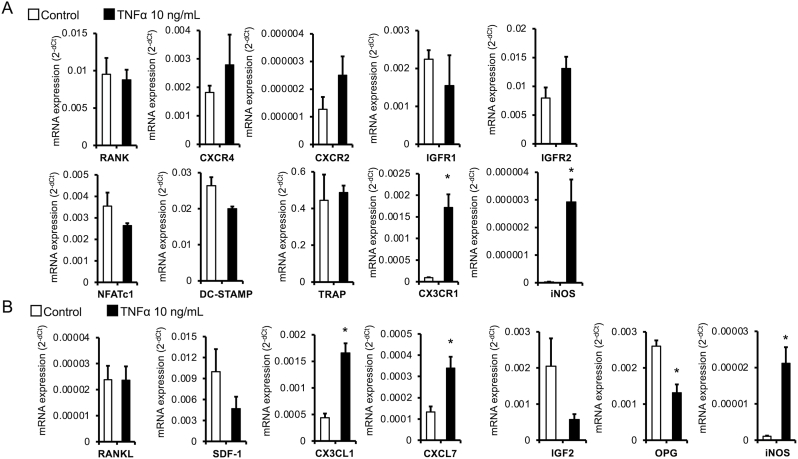
**TNFα treatment in the later stage of culture increased CX3CL1 and CXCL7 mRNA expression levels in non-adherent bone marrow cells.** Cultured bone marrow cells were treated with 100 ng/mL RANKL and 20 ng/mL M-CSF for 9 days. TNFα (10 ng/mL) was also added in the late (days 7–9) stage of cell culture. Cells were separated into osteoclasts and non-adherent bone marrow cells using the pronase procedure. RANK, CXCR4, CXCR2, IGF1R, NFATc1, DC-STAMP, TRAP, CX3CR1, and iNOS mRNA expression levels in osteoclasts (A) and RANKL, SDF1, CXCL1, CXCL7, IGF2, OPG, and iNOS mRNA expression levels in non-adherent bone marrow cells were quantified using quantitative RT-PCR. mRNA expression levels were normalized to that of β-actin mRNA. n = 3 per group, ∗*p* < 0.05 compared with the control (without TNFα stimulation).

To clarify the molecular mechanism underlying the induction of osteoclast differentiation by TNFα in the later stage of cell culture, we further examined expression levels of mRNAs related to osteoclast differentiation using quantitative RT-PCR. The analysis revealed that TNFα stimulation in the later stage of cell culture strongly induced the mRNA expression of iNOS, an enzyme involved in inflammatory processes, in both osteoclasts and non-adherent bone marrow cells (Fig. 3A and B). In non-adherent bone marrow cells, TNFα stimulation significantly increased mRNA expression of the chemokines CX3CL1 and CXCL7, which are potent chemoattractants and adhesion molecules, compared with cells not stimulated with TNFα (Fig. 3B). Furthermore, in osteoclasts, TNFα stimulation significantly increased the expression of CX3CR1, a receptor for CX3CL1, compared with osteoclasts not stimulated with TNFα (Fig. 3A). The mRNA expression of CXCR2, a receptor for CXCL7, did not change in TNFα-treated osteoclasts (Fig. 3A). By contrast, the mRNA expression level of OPG, which acts as a decoy receptor for RANKL and prevents it from binding to RANK, was significantly decreased by TNFα stimulation in non-adherent bone marrow cells. The mRNA expression levels of the osteoclast differentiation–related genes NFATc1 (a master transcription factor for osteoclastogenesis), DC-STAMP (an essential factor for osteoclast fusion), and TRAP did not change in osteoclasts in the presence of TNFα (Fig. 3A).

### 3.4 TNFR1-and TNFR2-neutralizing antibodies reduced the enhancement of osteoclast formation by TNFα treatment in the later stage of cell culture

**Fig. 4:**
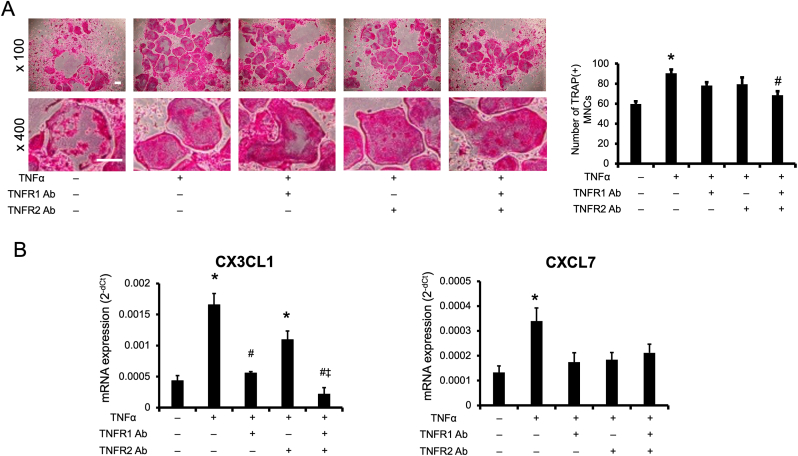
**TNFR1-and TNFR2-neutralizing antibodies reduced the enhancement of osteoclast formation by TNFα treatment in the later stage of cell culture.** (A) Cultured bone marrow cells were treated with 100 ng/mL RANKL and 20 ng/mL M-CSF for 9 days. TNFα (10 ng/mL) was also added in the late (days 7–9) stage of cell culture. Cells were also treated with anti-TNFR1 and/or anti-TNFR2 antibody (Ab) (each 3 μg/mL) 30 min before TNFα treatment. Cells were assessed by TRAP staining, and TRAP[+] MNCs were counted. n = 4 per group, ∗*p* < 0.05 compared with the control (without TNFα and without anti-TNFR1 and/or anti-TNFR2 antibody stimulation). ^#^*p* < 0.05 compared with TNFα alone. Images are shown at low ( × 100) and high ( × 400) magnification to visualize the overall cell density and detailed morphology of osteoclasts, respectively. Scale bars = 0.25 mm. (B) CX3CL1 and CXCL7 mRNA expression levels in non-adherent bone marrow cells were quantified using quantitative RT-PCR. mRNA expression levels were normalized to that of β-actin mRNA. n = 3 per group, ∗*p* < 0.05 compared with the control (without TNFα stimulation).

Finally, we added TNFα receptor–neutralizing antibodies to mouse bone marrow cell cultures to examine the role of TNFα receptors in the expression of CX3CL1 and CXCL7 induced by TNFα treatment in the later stage of cell culture. The findings of this experiment provide crucial insights into how TNFα affects the maturation process of osteoclast precursor cells, adding valuable context for understanding the mechanisms involved. A previous study showed that TNFR1 plays a significant role in osteoclast formation in cultured bone marrow cells [40]. Treatment with TNFR1-or TNFR2-neutralizing antibody alone had no effect on osteoclast formation, but simultaneous treatment with both antibodies significantly reduced osteoclast formation (Fig. 4A). With regard to the mRNA expression levels of CX3CL1 in non-adherent bone marrow cells, treatment with TNFR1-or TNFR2-neutralizing antibody alone significantly reduced the level of CX3CL1 mRNA expression. Additionally, simultaneous stimulation with both antibodies strongly reduced the expression level of CX3CL1 mRNA in non-adherent bone marrow cells, whereas the expression level of CXCL7 mRNA did not significantly change (Fig. 4B).

## 4 Discussion

In this study, we demonstrated that the addition of TNFα together with RANKL and M-CSF inhibits osteoclast formation, whereas TNFα stimulation initiated 7 days after pre-stimulation with RANKL and M-CSF induces osteoclast formation. Furthermore, the TRAP-positive area in cells treated with TNFα beginning in the later stage of cell culture was larger than that in cells without TNFα stimulation. The size of osteoclasts is reportedly correlated with their bone resorption activity [41]. Therefore, it seems likely that the presence of TNFα during the late maturation of osteoclasts enhances their bone resorption activity. Several studies have reported that the simultaneous addition of TNFα and RANKL inhibits osteoclast formation [35,37,42]. These data led us to speculate that circulating monocytes located distant from bone that have not begun to differentiate into osteoclasts may be insensitive to TNFα and inhibit osteoclastogenesis. This hypothesis would suggest that TNFα plays a significant role in the maturation process of osteoclast precursor cells induced to differentiate into osteoclasts by RANKL stimulation [42,43]. In this study, bone marrow cells were cultured with replacement of only half of the medium to prevent the loss of cell-derived components released into the culture environment. As shown in Fig. 2, endogenous TNFα, which is not derived from osteoclasts, may accumulate in the medium during the later stages of culture. The data also suggested that TNFα may have induced osteoclast formation due to the formation of a specific microenvironment in the medium during the later stages of osteoclast differentiation.

To examine the molecular mechanism of osteoclastogenesis induced by TNFα stimulation, gene expression in osteoclasts and non-adherent bone marrow cells was analyzed using quantitative RT-PCR. In non-adherent bone marrow cells, mRNA expression levels of CX3CL1 and CXCL7 were upregulated by TNFα stimulation. Generally, TNFα stimulation upregulates CX3CL1 expression in human fibroblasts [44]. Our previous study showed that SDF1, CX3CL1, CXCL7, and IGF2 are expressed at higher levels in non-adherent bone marrow cells and induce osteoclastogenesis [45,46]. In the present study, we also found that CX3CR1 mRNA expression was significantly increased in osteoclasts, whereas the expression of CXCR2, CXCR4, and IGF2R mRNAs remained unchanged. These results thus suggest that CX3CL1-CX3CR1 signaling plays an important role in TNFα-induced osteoclastogenesis. A previous report showed that RANKL inhibits CX3CR1 expression in macrophages during the initial 5 days of culture [47]. This could be one reason why TNFα did not promote osteoclastogenesis early in culture in the present study. Importantly, the addition of TNFα to cultures of non-adherent bone marrow cells reduced the expression in of OPG, which inhibits RANKL as a decoy receptor. This suggests that TNFα alters the environment to enhance RANKL activity in osteoclastogenesis.

We also investigated the involvement of TNF receptors in osteoclastogenesis and TNFα-induced chemokine expression. Treatment with a neutralizing antibody against either TNFR1 or TNFR2 alone did not affect osteoclastogenesis. However, simultaneous treatment with both antibodies significantly inhibited osteoclast formation. The lack of an osteoclast-suppressive effect by treatment with anti-TNFR1 or anti-TNFR2 antibody alone may be attributed to a differential contribution to osteoclastogenesis by TNFR1 signaling and TNFR2 signaling, similar to the suppression of CX3CL1 expression. TNFα may precisely regulate osteoclastogenesis by acting both to promote and inhibit osteoclastogenesis via TNFR1 and TNFR2 signaling. More detailed examinations of the microenvironmental changes resulting from differences in the timing of TNFα treatment are warranted.

This study has two notable limitations. First, although TNFα treatment showed opposite effects in the early and late stages of osteoclast differentiation, we could not determine the reason for this difference. To address this discrepancy, future studies employing unbiased, high-throughput approaches would be invaluable. For instance, time-course transcriptomic analysis of both osteoclasts and non-adherent bone marrow cells could be used to map the dynamic gene expression changes that define this shift. This could be complemented by a proteomic analysis of the secretome of non-adherent bone marrow cells to identify other key TNFα-regulated factors that contribute to the osteoclastogenic microenvironment. Second, in cells treated with anti-TNFR1 antibody alone, no significant changes in osteoclast formation were observed, but CX3CL1 expression was markedly decreased. However, osteoclast formation was significantly suppressed only in the group administered both the anti-TNFR1 and anti-TNFR2 antibodies. These results suggest that TNFα regulates osteoclastogenesis in a complex manner involving CX3CL1, OPG, and other factors. Functional studies employing specific inhibitors of CX3CL1 or CXCL7 signaling, or the use of recombinant proteins in the presence of TNFR blockade, could clarify the relative contributions of these chemokines to the observed phenotypes. Obtaining a precise understanding of these phenomena will require a comprehensive approach that analyzes not only the TNFα-induced changes in chemokine expression associated with osteoclast formation by non-adherent bone marrow cells but also the processes occurring within osteoclasts in response to TNFα stimulation.

In conclusion, our results strongly demonstrated that TNFα promotes osteoclast formation by upregulating the expression of CX3CL1 in non-adherent bone marrow cells in the late stage of osteoclast differentiation. Our findings underscore the critical importance of the temporal context of TNFα exposure and the dynamic interplay between developing osteoclasts and surrounding non-adherent bone marrow cells in determining the net effect on bone resorption. Understanding the stage-specific actions of TNFα could provide new perspectives for therapeutic interventions in diseases characterized by excessive osteoclast activity, such as rheumatoid arthritis. Rather than solely relying on systemic TNFα blockade, future strategies might explore more nuanced approaches. For example, selectively targeting downstream mediators such as the CX3CL1-CX3CR1 axis or developing interventions that specifically modulate TNFα receptor signaling or its effects on non-adherent bone marrow cells during critical windows of osteoclast maturation within inflamed joints could offer a more refined way to control pathological bone resorption while potentially minimizing the systemic side effects associated with broad TNFα inhibition. Further research is warranted to translate these mechanistic insights into the identification of viable therapeutic targets.

## Ethical approval for experiments with animal

All animal experiments were approved by the Institutional Animal Care and Use Committees of Nagoya City University (approval no.: H29–P-01).

## CRediT authorship contribution statement

**Yuto Otsuka:** Writing – original draft, Visualization, Methodology, Investigation, Funding acquisition, Formal analysis, Data curation, Conceptualization. **Narumi Hattori:** Methodology, Investigation, Formal analysis, Data curation, Conceptualization. **Hiromasa Aoki:** Writing – original draft, Methodology, Investigation, Funding acquisition, Formal analysis, Data curation, Conceptualization. **Kohki Toriuchi:** Writing – original draft, Formal analysis, Data curation, Conceptualization. **Yasumichi Inoue:** Resources, Formal analysis. **Hidetoshi Hayashi:** Resources, Formal analysis. **Gen Kuroyanagi:** Writing – original draft, Formal analysis. **Yohei Kawaguchi:** Funding acquisition, Formal analysis, Conceptualization. **Yuko Waguri-Nagaya:** Funding acquisition, Formal analysis, Conceptualization. **Mineyoshi Aoyama:** Writing – review & editing, Supervision, Project administration, Funding acquisition, Formal analysis, Conceptualization.

## Declaration of generative AI and AI-assisted technologies in the writing process

During the preparation of this work, the authors used Gemini (Large Language Model by Google) to improve the readability, clarity, and conciseness of the manuscript. After using this tool/service, the authors reviewed and edited the content as needed and take full responsibility for the content of the published article.

## Funding

This work was supported in part by 10.13039/501100001691Grants-in-Aid for Scientific Research from the Japan Society for the Promotion of Science, KAKEN grant numbers 20K22715, 19K18473, 20K09465, and 20K08211. This work was also supported by 10.13039/501100025019JST SPRING, grant number JPMJSP2130.

## Declaration of competing interest

The authors declare that they have no known competing financial interests or personal relationships that could have appeared to influence the work reported in this paper.

## Data Availability

Data will be made available on request
